# Examining how study teams manage different viewpoints and priorities in patient‐centered outcomes research: Results of an embedded multiple case study

**DOI:** 10.1111/hex.13765

**Published:** 2023-05-30

**Authors:** Maureen E. Maurer, Tandrea Hilliard‐Boone, Karen Frazier, Laura Forsythe, Rachel Mosbacher, Kristin L. Carman

**Affiliations:** ^1^ American Institutes for Research (AIR) Chapel Hill North Carolina USA; ^2^ Patient‐Centered Outcomes Research Institute (PCORI) Washington District of Columbia USA

**Keywords:** comparative effectiveness research, conflict resolution, consensus, patient and public involvement, patient and stakeholder engagement, patient‐centered outcomes research, team science

## Abstract

**Introduction:**

Limited evidence exists about which patient and stakeholder engagement practices support or hinder study teams as they negotiate different viewpoints in decisions about the design and conduct of patient‐centered outcomes research.

**Methods:**

We applied a multiple‐embedded descriptive case study design for six studies funded by the Patient‐Centered Outcomes Research Institute (PCORI). We interviewed 32 researchers and stakeholder partners, including patients, caregivers and clinicians, and reviewed documents related to each study (e.g., publications, and progress reports submitted to PCORI).

**Findings:**

Overall, researchers reported that incorporating different viewpoints was a strength or opportunity to learn rather than something to be avoided or dreaded. Across cases, different viewpoints and priorities, often related to ethical or pragmatic considerations, emerged between researchers and stakeholders, between stakeholder groups (e.g., patients and clinicians) or within groups (e.g., amongst researchers). Examples of navigating different viewpoints arose across study phases. The length of time to resolve issues depended on how strongly people disagreed and the perceived importance or impact of decisions on the study. All cases used collaborative decision‐making approaches, often described as consensus, throughout the study. Interviewees described consensus as using negotiation, compromise or working towards an agreeable decision. To encourage consensus, cases actively facilitated group discussions with an openness to diverse opinions, remained flexible and open to trying new things, referenced a ground rule or common goal and delegated decisions to partners or smaller workgroups. When viewpoints were not easily resolved, cases used different approaches to reach final decisions while maintaining relationships with partners, such as elevating decisions to leadership or agreeing to test out an approach. No one engagement structure (e.g., advisory group, coinvestigator) stood out as better able to manage different viewpoints. Teams adjusted engagement structures and behaviours to facilitate an overall culture of inclusion and respect. Partners acknowledged the intentional efforts of researchers to incorporate their perspectives, navigate challenges and communicate the value of partner input.

**Conclusion:**

By using collaborative decision‐making in the early stages and throughout the study, researchers built trust with partners so that when decisions were difficult to resolve, partners still felt listened to and that their input mattered.

**Patient or Public Contribution:**

Members of the PCORI Patient Engagement Advisory Panel in 2019–2020 provided input into the design of the study, including the research questions and approaches to data collection and analysis.

## 1 INTRODUCTION

Emerging literature documents the benefits of patient and other stakeholder engagement in research, including active influence by stakeholder partners on study protocols and study enrolment rates.^1^, ^2^, ^3^, ^4^, ^5^ These contributions have resulted in impacts on studies' acceptability, feasibility, rigour, relevance and even on the scope and quality of engagement.^6^, ^7^, ^8^, ^9^

Study teams may encounter challenges with engagement, such as building and maintaining relationships between researchers and partners, including group dynamics.^10^, ^11^ Effective collaboration on multistakeholder teams requires encouraging diverse perspectives while negotiating disagreements and conflict.^12^, ^13^, ^14^, ^15^, ^16^ Yet, researchers have voiced challenges in managing conflicting feedback when different perspectives arise during decision‐making.^17^, ^18^ Further, despite the growing practice and science of engagement, much of the understanding of practices that support negotiating different viewpoints in decision‐making has been based on publications that include narratives from single studies or studies with nominal information about such practices.^19^, ^20^

To advance our understanding of ways to manage diverse perspectives, we gathered and analyzed information within and across six case studies. Each case was a study team funded by the Patient‐Centered Outcomes Research Institute (PCORI). PCORI requires awardees to engage diverse stakeholders in comparative effectiveness research studies. PCORI provides broad guidance about how to engage partners,^21^ which allows study teams to explore practices and address challenges with their available resources and specific to their institutional and project contexts.^4^, ^6^, ^22^

The research questions were:

- How do study teams manage and balance different viewpoints and priorities when making decisions about study conduct?
- How do engagement practices—such as behaviours, interactions or structures—help or hinder the management of different viewpoints and priorities?

## 2 METHODS

We applied a multiple‐embedded descriptive case study design.^23^ This approach includes multiple units of analysis in each case (e.g., interviews with researchers and partners, document review) and improves validity through data source triangulation.^24^ We included six cases to compare and corroborate findings across cases.

PCORI's Patient Engagement Advisory Panel provided input to the design of the study, including the research questions and approaches to data collection and analysis. The American Institutes of Research Institutional Review Board reviewed this study.

### 2.1 Sample

We selected candidate cases from a purposeful sample of 58 PCORI‐funded projects that participated in a recent qualitative study on the influence and impact of engagement.^7^ The original study sample included 110 researchers and stakeholder partners from projects that were a heterogeneous representation of PCORI's portfolio regarding study completion status (i.e., complete and active), funding announcement category, PCORI priority content area, study design, study populations and health conditions.

Of the 58 projects, we excluded 16 where interviewees demonstrated difficulty with recall. We then identified 26 cases in which an interviewee described one or more of the following, indicating a difference in viewpoints or priorities:

- Deliberation between stakeholder values and scientific or technical requirements.
- Change in engagement structures over time.
- Disagreements or conflicts about a study decision or task.

For eligible cases, we created a blinded list of characteristics, including the rationale, level of engagement experience reported by the PI, types of stakeholder partners involved and project completion date. Five team members independently selected 12 cases for inclusion with the goal of achieving diversity across characteristics. Combining these lists resulted in 14 cases; the team identified 6 priority and 8 alternate cases.

### 2.2 Outreach and recruitment

We conducted email outreach to recruit PIs from identified cases between May 2020 and March 2021. Due to the onset of the COVID‐19 pandemic, we offered flexible scheduling. We emailed the PI to assess interest, even if immediate participation was not possible. We then scheduled a call to discuss the case study approach, the process for selecting interview participants, how PCORI will use the case study results and concerns about confidentiality or compensation.

Projects were enroled as a case study if the PI was willing to participate in an interview and follow‐up activities such as reviewing case narratives, and providing contact information for up to five study team members (researchers, partners) to participate in interviews. After obtaining a list of recommended participants from the PI, we contacted those individuals via email or phone to assess their interest and availability.

### 2.3 Data collection

The data collection and analysis team originally included the project lead (M. E. M.) and three analysts (K. F., T. H.‐B. and R. Ma.). All had prior training and experience conducting qualitative research, including the original study on influence and impact, and were familiar with engagement in research.

For each case, one analyst conducted a document review and interviews. Documents included interim progress reports submitted to PCORI, publications and interview transcripts and analysis memos from the original study. The analyst then extracted information about engagement structures and practices, relationships and interactions and challenges encountered.

Based on the document review, analysts developed tailored interview protocols for each case. All interviews used a core set of questions: (1) How did you make decisions when people disagreed? (2) Were there times when you had to balance competing interests? (3) How did you or others in the project go about making people feel welcome? Heard? Respected? (4) How did/have you maintained relationships amongst team members, including partners, over time?

Interviews lasted up to 60 min and took place via Zoom—either video or phone only. We conducted interviews on a rolling basis between August 2020 and April 2021. Partners were interviewed individually; some researchers participated in joint interviews for convenience. Participants received a gift card for $125. All interviews were audio‐recorded and professionally transcribed.

### 2.4 Data analysis

For within‐case analysis, analysts first extracted data from individual interviews into a structured memo with standard embedded case study topics, including project characteristics and background information (e.g., number and type of stakeholders, engagement structures), how projects managed and balanced different viewpoints and priorities and how engagement practices helped or hindered balancing or managing different viewpoints and priorities. For five cases, the same analyst (K. F., T. H.‐B.) assigned to data collection conducted the analysis; in one case, a new analyst (Z. S.) conducted the analysis.

Analysts developed a 5–10‐page case narrative that concisely described the project's experience with balancing differing viewpoints. The project lead (M. E. M.) and team members, including PCORI staff (R. Ma., K. L. C., L. F. and R. Mo.) reviewed each case narrative for clarity and relevance to the research questions. We shared this narrative with the PI for member‐checking to confirm findings and incorporated any edits or clarifications.

After completing the within‐case analyses, the project lead (M. E. M.) and two analysts (K. F. and T. H.‐B.) drafted a memo with matrices to systematically categorize the reported occurrence of different viewpoints and priorities across study activities, decision approaches across cases, engagement structures and reported engagement behaviours and strategies. The memo also reviewed conclusions across each case to understand common versus unique ways to manage different viewpoints and priorities. The entire team discussed initial findings and determined areas for further examination. We then discussed and iteratively synthesized findings in three areas: different viewpoints and priorities encountered, decision‐making processes and engagement structures and behaviours.

Throughout the study, the team met weekly to ensure a consistent approach across cases, discuss emerging findings, share personal reflections on how the data aligned with or differed from expectations and test assumptions, biases and conclusions.

## 3 FINDINGS

We reached out to 11 PIs: 6 agreed to participate, 2 declined, and 3 did not respond to initial or follow‐up email requests. Across the six cases, we conducted 28 interviews with 32 interviewees, including 6 PIs, 13 other researchers and 13 partners. Partners included patients, caregivers, parents, clinicians and a healthcare administrator. We did not collect demographic information for interview participants. Table 1 provides an overview of the final six cases.

**Table 1:** Characteristics of each case study.

Feature	Case 1, communication matters	Case 2, building bridges	Case 3, be humble	Case 4, fostering inclusion	Case 5, let's give it a shot	Case 6, we're in this together
Rationale for selection that indicated different viewpoints or priorities	Change in engagement structure	Deliberation between stakeholder values and scientific requirements	Deliberation between stakeholder values and scientific requirements	Change in engagement structure	Disagreement or conflict	Disagreement or conflict
Number of interviews completed	5 interviews: 1 PI, 3 research team, members, 1 partner	4 interviews: 1 PI, 2 engagement coordinators, 1 partner	4 interviews: 1 PI, 3 partners	5 interviews: 7 project leaders including PI, 2 partners	5 interviews: 1 PI, 1 researcher, 3 partners	5 interviews: 1 PI, 1 researcher, 3 partners
PCORI funding priority area	Improving healthcare systems	Addressing disparities	Improving healthcare systems	Communication and dissemination	Improving healthcare systems	Improving healthcare systems
Study design	Prospective and retrospective cohort	Randomized controlled trial (RCT)	RCT, quasi‐experimental	RCT, quasi‐experimental	RCT	RCT
Stakeholders engaged	Patients, caregivers, providers, payers, policymakers	Community clinic, parents, teens	Patients, caregivers, clinicians, system and public health reps, payers, policymakers	Physicians, nurses, parents, physician educators	Nurses, clinicians, patients, administration, consultants	Patients, caregivers, advocacy organizations, clinicians, hospitals, payers, policymakers, trainers
Engagement structure	8 partner co‐investigators; 24‐member Stakeholder Advisory Group	Clinic partners on leadership team; parent and teen advisory groups (held in Spanish)	20‐plus member Stakeholder Advisory Group; study task‐related working groups	Nurse advisory board; family advisory board; study task‐related workgroups with all stakeholders	Partner co‐investigator; core workgroup; patient and family advisory council	10‐member Executive Committee

Abbreviation: PCORI, Patient‐Centered Outcomes Research Institute.

Below, we summarize each case and present findings from the cross‐case analysis.

### 3.1 Within‐case analysis

Overall, interview participants within each case reported similar accounts of what had happened during the original study.

#### 3.1.1 Case 1: Communication matters

After a rocky start in which partners felt disengaged, the study team pivoted their engagement approach to include the Stakeholder Advisory Group (SAG) in study decisions. The diverse, geographically dispersed 24‐member SAG included individuals with many diverse perspectives, including patients, family caregivers, providers, payers, advocacy organizations and policymakers with lived and professional experience in health care. The SAG's charge was to help keep the project focused on the needs of all stakeholders. The initial engagement plan involved quarterly meetings with the SAG via conference calls facilitated by the PI, quarterly updates to resources via a shared online portal and one annual in‐person meeting with the full project team. Within the first 6 months, the team realized that this infrequent and largely passive communication approach did not work well to meaningfully engage SAG members. Given the complexity of the project, checking in with the SAG after months of activity created challenges. Many SAG stakeholders said it made them feel disengaged from the work primarily due to the time lag between the quarterly meetings and the density of information covered during them. Stakeholders wanted more frequent involvement with the research team so that they could contribute to decision‐making about the study conduct. They also wanted more hands‐on responsibility for the engagement itself and more ways to engage with the team instead of reading materials on the online portal. The team pivoted to a new design that placed stakeholders in charge of their own group by bringing on one patient and one family caregiver from the core research team as co‐chairs of the SAG, which created formal linkages between the research team and the stakeholder group. The team also invited the SAG to attend one research team meeting each month, distributed a monthly newsletter with SAG member spotlights, hosted more inclusive annual in‐person meetings and administered experience surveys to identify ways to improve engagement. This new structure created abundant opportunities for stakeholders and researchers to exchange views and shape decisions that took different perspectives into account. Formal and informal feedback loops also allowed the research team to explain when and why decisions did or did not integrate stakeholder viewpoints.

#### 3.1.2 Case 2: Building bridges

A gradual process of relationship building resulted in strong partnerships between researchers, clinic leadership and parents. Before the study began, the PI spent 6 months developing relationships with partners, starting with a local clinic that served patients whose first language was Spanish. The PI first invited the clinic's director to a meal and a ‘friendly chat’—an informal conversation to exchange ideas and explore common ground—at a restaurant near the clinic. Before becoming involved in this study, the clinic ‘felt burned out’ by research because of previous bad experiences. Through ongoing conversations, the clinic staff taught the PI and the broader research team about the clinic—what its values were, how it operated and what it needed—and the researchers listened. During this process, the PI worked with the clinic director to plan the study and prepare the PCORI application in a way that would be feasible and mutually beneficial. The clinic also connected the PI with parents, who helped write the proposal. After receiving the PCORI award, the research team—which included clinic leadership and the parent advisors—set out to develop a parent advisory group to help plan the study's intervention and dissemination. Partway through the study, the team received additional funds to collect data from adolescents and formed a second advisory group of adolescent partners. Due to the limited interaction in adolescent care, this second advisory group membership was not as consistent. Throughout the study, the researchers worked to develop and nurture trusting, respectful relationships with each group. The researchers did so by (1) showing respect to their partners and their partner's needs as they listened and acted on what they heard from clinic staff, (2) establishing effective communication patterns with their partners as they included the clinic in conversations, decision making, and regular meetings and (3) maintaining a healthy perspective about different viewpoints—that they are to be expected and can be negotiated.

#### 3.1.3 Case 3: Be humble

With a ground rule of “we are on the same team*”*, the PI encouraged a ‘different method of doing research’ focused on the free exchange of ideas and collective ownership of the study. The resulting environment supported open discussions of differing perspectives during decision‐making. The PI's approach comprised three main strategies: (1) setting the tone with the ground rule, (2) putting humility into practice by expressing genuine curiosity, asking open‐ended questions, and frequently emphasizing that stakeholders' opinions matter and (3) encouraging stakeholders to take on an active role in the design and conduct of the study by participating in decision making in study task‐specific working groups. The project had a 20‐plus member SAG and the working groups, such as for recruitment or communication with enroled study participants, had decision‐making authority over these tasks. The team intentionally included at least one parent and one clinician partner in each working group to have partner voices represented in every decision‐making body. The team worked towards consensus in decisions and prioritized stakeholders, especially parent partners, feedback on decisions. Stakeholder perspectives were integrated into three key decisions about the study design: revising the original randomized study design to an alternate design that met researchers' requirements for scientific rigour, adding a patient‐centered outcome and expanding the study inclusion criteria.

#### 3.1.4 Case 4: Fostering inclusion

After recognizing what patient partners could offer early in the project, the team modified the engagement approach to better integrate their family and nurse partners into study decisions. Initially included as separate advisors, the team made sure family and nurse partners had roles in workgroups and study leadership. In this way, partners had more direct participation in task decision‐making. The researchers also maintained family and nurse advisory councils as separate groups. Decisions were made at the workgroup level, and the team used a consensus‐building decision‐making approach throughout the study. At times, the team struggled to engage certain stakeholder groups on the project team, including nurse partners and parent partners with diverse lived experiences, including fathers and Latino or African American parents. Even with the challenges of partnering with diverse voices, the team effectively demonstrated that they valued their partners' perspectives by including them in the decision‐making process, listening to and respecting different viewpoints and prioritizing responsiveness to partners. This meant that the active partners—both nurses and parent partners—felt that the researchers valued everyone who was participating.

#### 3.1.5 Case 5: Let's give it a shot

When perspectives differed on how to redesign the intervention, the team agreed to move forward with an approach and ‘give it a shot’. Before receiving PCORI funding, a rehabilitation centre planned to overhaul its patient self‐care curriculum to an evidence‐based peer‐led intervention so that peers would teach classes rather than nurse educators. The team formed a workgroup, and after receiving funding, this workgroup was responsible for all study decisions. Members included the PI, a research scientist who was also a clinician, two peer supporters, nurse educators, therapists and an administrator. All members shared the goal to help improve the health of patients at their centre. However, when learning about plans to shift to peer‐led intervention, nurse educators were hesitant; they believed patients needed to learn essential information about managing their condition from clinicians. After several discussions, the group proposed, and the nurse educators reluctantly agreed, to ‘give it a shot’ by testing part of the peer‐led intervention and collecting preliminary data about its effectiveness. Coming up with an approach to test the peer‐led curriculum on a small scale demonstrated respect for all perspectives. After witnessing the success of the peer‐led intervention, the nurse partners became advocates for it. The workgroup approached consensus‐building through ‘constant discussion’ with one another by sharing ideas, reacting, and offering suggestions for developing and carrying out the interventions. They also made their meetings a welcoming and comfortable place—an informal lounge area with food provided where they could sit on couches, talk, and laugh together—to support relationship building.

#### 3.1.6 Case 6: We're in this together

Using a collectivist approach in which project goals and interests were prioritized over those of individuals, the team navigated study challenges related to providing training and study participant enrolment. The co‐PIs established a 10‐member Executive Committee to help guide the project. The Executive Committee included physicians in primary and psychiatric care, peer recovery specialists, executive leaders from health systems and human and social services agencies and two members with lived experience. The team also engaged community outreach partners who were responsible for enroling patients into the study, hiring and managing community health workers and facilitating peer groups. Team members agreed that although there were some intense meetings, they were viewed as problems that the team came together to solve, rather than major disagreements. For example, community outreach partners and researchers had different perspectives about approaches to enrolment (e.g., frequency of contact attempts with prospective recruits) and worked to find compromises that balanced the needs of the project with the needs of the community members. Although hitting enrolment targets was important, community outreach partners were also mindful of potential enrollees' receptivity to active recruitment strategies. The team's collectivist approach, which kept the goals of the project first and encouraged open sharing and feedback, and involved consensus‐based decision‐making, helped the team identify solutions that took everyone's perspective into consideration and pursue a balanced approach.

### 3.2 Cross‐case analysis

We describe themes across cases in three main areas: different viewpoints and priorities encountered, decision‐making processes and engagement structures and behaviours.

#### 3.2.1 Different viewpoints and priorities encountered

Different viewpoints and priorities emerged between researchers and stakeholders, between stakeholder groups (e.g., patients and clinicians), or within groups (e.g., within a stakeholder group or amongst researchers), often related to ethical or pragmatic considerations. Patients and caregivers often focused on what they thought would result in the best outcomes for study participants, even when disagreeing with each other about approaches to do so. Clinicians focused on their ethical responsibilities to patients. Researchers focused on maintaining study rigour to yield the highest quality evidence, debating with each other about approaches to do so. Pragmatic considerations included implementing the intervention or collecting data in ways that worked for patients and clinicians. Examples of when study teams had different viewpoints and priorities typically occurred in the early phases of the study, when teams were planning approaches to the study design or intervention implementation, or when studies encountered problems, such as with recruitment or enrolment. Researchers were often the gatekeepers in eliciting, managing and incorporating the different viewpoints.

Although interviewees in three cases used the term ‘conflict’ to describe different viewpoints and priorities [1, 4, 6], most interviewees across cases did not. Instead, interviewees used terms like disagreements, debate or discussion, strong or big personalities, pushback or resistance and others.

The length of time to resolve differing viewpoints appeared to depend on how strongly people disagreed, the nature of the decision in terms of the perceived importance or potential impact on the study, and whether a decision required PCORI approval. For example, in case 3, when examining inclusion criteria for study participants, some stakeholders expressed that teenagers, not just preteens, could benefit from the intervention. Researchers noted that the intervention had not previously been studied with teenagers but did not express serious opposition to expanding the age criteria. After a brief discussion, researchers integrated the suggestion into the study design. Another change to the study design in case 3 took longer to implement. During the kickoff meeting, many parents and some clinicians objected to randomizing participants to usual care given that existing evidence strongly supported the intervention's efficacy. The PI and research team recognized the need to integrate the stakeholders' perspective to give all study participants access to effective intervention with the research team's view on the need for a high degree of scientific rigour. Stakeholders understood the importance of maintaining scientific rigour and respected the researchers' desire to do so. They acknowledged that making changes to a study ‘wasn't just [as] simple [as] saying “hey, yeah, let's switch”’. Researchers immediately started deliberations amongst themselves about ‘different study designs, different pragmatic study designs, and how to implement the study with stakeholders’. Once researchers found an acceptable design that met their requirements for scientific rigour, they consulted with PCORI staff, who supported the change. In this new design, all study participants used the intervention, but some received an additional, small‐scale intervention designed to encourage the use of the baseline intervention. Researchers presented the new design to the stakeholders. The entire process of changing the study design took about one month. Stakeholders applauded the decision and the quick amount of time it took to make.

In contrast to other cases, case 2 described managing different viewpoints and priorities as a foundational approach to the study versus something that happened in individual decisions.

There were millions of instances…We make ongoing decisions about implementation. Every decision point was a point where something wasn't working well, and we made some slight change to fix it… We just kept working to find alternatives… We tried to continue with the goal, the common sort of shared notion of what we wanted but do it a different way. (Case 2, PI)

In almost all cases, researchers reported that having and incorporating different viewpoints was a strength and opportunity to learn rather than something to be avoided. For example, one PI said:

I strongly encourage people to speak their opinions, especially if they're divergent with what it seems everybody else is thinking because that's the only way we're going to learn and grow… That was a nice aspect of the entire project. It strengthened it. (Case 1, PI)

As a result, the representation of diverse viewpoints was important. However, two cases [4, 6] described challenges with the representation of diverse stakeholder perspectives on their teams. For example, case 4 had difficulty identifying and maintaining relationships with fathers and parent partners from diverse racial and ethnic backgrounds. Consequently, their family advisory council was largely comprised of White, educated, affluent mothers who had an existing connection to someone on the research team. A researcher said: ‘We ended up with a lot of well‐to‐do middle‐aged White women who were involved…they had the means and capability. In terms of thinking about diversity, it's been a challenge for us’.

#### 3.2.2 Decision‐making processes

All cases reported using collaborative approaches to decision‐making, which interview participants often described as reaching consensus. Although interview participants were not asked to explicitly define consensus, cases generally described consensus as conversations using negotiation, compromise or working towards an agreeable decision. One partner summarized the process as the team would:

Work the problem until we came to consensus…There was no voting or someone's going to break a tie – none of that. It was really conversation [about] what are the pros and cons? What are the risks and benefits of doing this? (Case 4, Partner)

In five cases [1, 2, 3, 4, 6], interviewees discussed ways they incorporated—and sometimes prioritized—patient partner feedback in this decision‐making process. In three of these cases [2, 3, 6], researchers described incorporating patient partner feedback in decisions where researchers were hesitant or typically would not have made the suggested changes. For example, in case 6, the team listened to a patient stakeholder who felt strongly about making changes to the survey. A researcher said:

[A stakeholder] had things to say in terms of how we asked questions and what to ask and what was ethical and what wasn't ethical. That was really important for us. We listened. We really changed a lot of things because of his point of view. (Case 6, Researcher)

Cases reported using a variety of strategies to help encourage and facilitate consensus across different viewpoints, including actively facilitating group discussions with an openness to diverse opinions; remaining flexible and open to change; having a ground rule or common goal to reference; and giving partners or smaller workgroups authority to make independent decisions.

No cases reported using a formal voting process, defining what consensus meant with study teams at the start of the study, or agreeing to a preset approach if consensus could not be reached.

When viewpoints were not easily resolved, cases used different approaches to reach final decisions, such as elevating decisions to leadership or agreeing to test out an approach. For example, in case 5, after several discussions, everyone but the nurse educators initially agreed to try out a peer‐led curriculum. In the meeting where this decision was made, the workgroup—as a whole—communicated to the nurse educators that ‘we understand that you might have a differing opinion, but we're moving forward with it because it [is] evidence‐based’. The group, including the nurse educators, agreed to test the peer‐led and nurse‐led interventions for one curriculum topic. They video‐recorded test classes using both approaches and observed how many patients were actively participating (e.g., nodding in response to the instructor, answering questions, or raising their hands) versus not actively participating (e.g., sleeping, using their phones, or otherwise distracted). They found that patients were overwhelmingly more active in peer‐led education classes and more socially engaged with their peers than they were with nurse educators. After witnessing its success, the nurse partners became one of the intervention's biggest advocates.

In a couple of instances when interviewees reported major conflict‐either resolved or unresolved, it initially resulted in discontent [1] or hurt feelings [5] amongst partners. In case 1, researchers acknowledged the conflict and changed the engagement structure to expand opportunities for partners to contribute. In case 5, the lead nurse partner expressed concerns that the group had not treated them as valuable or important to decision‐making, and a researcher apologized for the hurt feelings [5]. A researcher said:

It wasn't like somebody was the bad guy here. Everybody had the same goal… to provide the best care to the patient. That was always front and center. When that happens and then you start liking each other in the process and you start talking about how you can do things better and differently, and basically apologize for what happened, that you didn't recognize their importance at the very beginning, [relationships] started to evolve. (Case 5, Researcher)

Although all cases described including stakeholder partner input in decisions generally, three cases reported a specific decision where stakeholder input was not incorporated or that appeared to be nonnegotiable [2, 5, 6]. These decisions focused on study design rigour [2, 5] or fidelity to an evidence‐based intervention [6]. Similar to case 3, parent partners in case 2 had wanted all patients to access the intervention. However, without a viable alternative, the PI did not want to change the study design from a randomized controlled trial to maintain scientific rigour. The researchers explained why randomization would provide the strongest evidence for the intervention, and the partners understood this reasoning. Also, because researchers had built trust with partners over time and had previously incorporated partner input into decisions, partners still felt heard and valued.

#### 3.2.3 Engagement structures and behaviours

No one engagement structure emerged as better able to help or hinder management of different viewpoints. All but one case [6] used multiple types of engagement structures (e.g., co‐investigator, advisory group, working groups/committees), and all cases also worked with single‐ or multistakeholder advisory groups. All cases had stakeholder partners in roles with decision‐making authority, either as co‐investigators, on the core research team, or as part of working groups.

Study teams worked within each type of structure and adjusted when needed to better include diverse viewpoints in decisions. For example, in case 1, the team made major adjustments to the structure by putting partners in leadership roles for the advisory group. They also implemented new approaches for open and inclusive communication, such as inviting partners to team meetings and distributing a monthly newsletter. The PI said:

[The stakeholder advisory group] brought up that they felt disengaged from the project…We ended up inviting them to our research calls…What was nice was that they actually contributed a lot on those research calls. (Case 1, PI)

Similarly, in case 4, the team realized that, with a single SAG, they were missing an opportunity to involve patients in decision‐making and subsequently adapted their structure to include patients in each of the study's workgroups. Further, in case 6, the team made minor accommodations to better include the two members with lived experience in decision‐making, for example, by reaching out after the meeting. However, in hindsight, researchers recognized how other Executive Committee members often dominated the discussions, which may have limited the engagement in the study.

Behaviours to help manage different viewpoints and priorities focused on inclusion, creating a respectful and welcoming environment and open and intentional communication. Table 2 highlights behaviours that interviewees reported to help manage different viewpoints and priorities.

**Table 2:** Engagement behaviours related to managing different viewpoints and priorities.

Category	Behaviours (number of cases reported)
Inclusion	Creating meaningful opportunities for partners to contribute Included partners in decision‐making processes and on leadership teams (6) Worked to integrate multiple perspectives in decisions (5) Encouraged stakeholders to take active roles in the study (2) Invited partners to participate in working groups to ensure all perspectives were represented (2) Shifted engagement structure to be more inclusive (2) Crafted specific questions for stakeholder advisory group meetings to get focused input (2) Used a gradual process of conversations to build trust and define the project in a way that is mutually beneficial (1) Invited stakeholders to research team calls (1) Provided childcare during meetings (1) Scheduled meetings at a time and location convenient to stakeholders (1) Eliciting and synthesizing diverse viewpoints Asked stakeholders for their views and input during meetings (6) Used active facilitation to elicit views from all stakeholders (6) Built‐in an open forum for group discussions in agenda (3) Reiterated the importance of different viewpoints (3) Managed to dominate voices during discussion (3) Asked open‐ended questions that expressed genuine curiosity (3) Identified other ways to get input from quiet, intimidated, or shy stakeholders, such as following up one‐on‐one after meetings or getting written feedback (3) Used teach‐back to elicit and then summarize perspectives and decisions (1) Administered experience surveys (1)
Creating a respectful and welcoming environment	Setting a tone of respect and collaboration Remained open to change (6) Listened and responded to stakeholder perspectives (6) Had members of the research team who served as cultural bridges or liaisons (2) Set expectations for stakeholder participation (2) Sent meeting agenda to stakeholder advisory group members in advance (2) Used first names to diminish hierarchy (1) Set tone by setting and repeating the ground rule that we are all equal on the team (1) Focused on what was best for the project rather than individual perspectives (1) Developed a shared mental model to get everyone on the same page during study objectives (1) Making interactions informal and fun Had meetings in a comfortable space and provided food (2) Participated in rituals of celebration, such as life events, birthdays or holidays (2) Included time for informal conversations during meetings (2) Held retreat at a family‐friendly resort (1) Planned informal dinners (1) Used icebreakers to increase comfort (1)
Open and intentional communication	Had feedback loops from researchers to partners about how input was applied (5) Maintained open communication (4) Conducted one‐one‐one outreach to stakeholders (3) Reported being transparent about study decisions and activities (2) Acknowledged or apologized for unintended harm, such as discontent or hurt feelings (2) Shifted communication approach to be more inclusive, which included distributing a newsletter and designating a point person (1)

Before managing different viewpoints and priorities, teams had to first invite and elicit diverse viewpoints. These inclusion behaviours related to both creating opportunities for partners as well as eliciting and synthesizing diverse viewpoints in ways that worked for their team and study. Summarizing these types of behaviours, one partner said:

They always asked our opinion. Always. Even at the very end when they were writing up the paper, they were like ‘hey, who wants to be a part of this, who wants to read it?’ They really wanted us to be involved, and that was obvious because they were always asking us to be involved. (Case 3, Stakeholder)

In a respectful and welcoming environment, partners felt that they had valuable expertise to share. These behaviours focused on setting a respectful and collaborative tone, such as remaining open to change and listening and responding to stakeholder perspectives. As one partner noted:

The way they respected us, the way they listened to us, they honored our time, took input, stopped to pause and say, ‘is this working?’ Those things then helped us to know it was a positive relationship and we could talk back to them and stay engaged. They weren't dismissive, they weren't just looking at deliverables and driving so hard at those that they were steamrolling all of us out in the community. (Case 2, Partner)

Behaviours related to open and intentional communication helped researchers and partners build trust, relationships, and a sense of community. Commonly reported behaviours were having feedback loops from researchers to partners about how input was applied and maintaining open communication. One researcher said:

We need to learn to be humble, to learn to do the research in different ways, to learn to include families' concern from the beginning, not late. And to learn to deal with them in two‐way communication, not one‐way. To have trust and to have transparency in the relationship with the stakeholders. (Case 3, Researcher)

## 4 DISCUSSION

These findings advance an understanding of how study teams perceive and manage different viewpoints and priorities that have implications for engagement practices researchers and partners can adopt.

Overall, study teams established a culture of inclusion and respect that helped them manage different viewpoints and priorities. The foundation to successful management for researchers may be viewing different viewpoints as a strength and opportunity to learn rather than a challenge to be avoided. Different viewpoints are a productive part of any research endeavour; in these six cases, engagement surfaced possible challenges early in the study so that teams could adjust approaches and identify solutions.

Another practice that facilitated the successful management of different viewpoints included being flexible in how to structure engagement, for example by adjusting approaches to better include partners in study decisions. Cases identified concrete behaviours focusing on inclusion, establishing trust and ongoing communication, that other study teams could adopt. Also, by using collaborative decision‐making approaches and including stakeholder input in decisions in the early stages and through the study, researchers were able to build trust with partners so that when decisions were difficult to resolve or were nonnegotiable, partners still felt valued. Further, as in case 4, other studies have documented challenges researchers face in identifying and engaging partners who represent diverse racial, ethnic, gender, educational and socioeconomic backgrounds.^25^, ^26^, ^27^ Although not an explicit focus of this study, these concrete behaviours related to inclusion, trust, and communication may facilitate engagement with historically excluded and marginalized communities.

All cases reported using a collaborative approach to decision‐making, often described as consensus. Interviewees' description of consensus reflects literature that posits consensus as a decision arrived at through collective discussion where group members have an opportunity to influence the decision rather than full and unanimous agreement, which may be more challenging to reach in real‐world settings with diverse stakeholders.^28^, ^29^ Further, without naming them directly, cases described examples of established participatory decision‐making models that were applied to different types of decisions,^29^, ^30^ such as:

- Team leader gathers input from the team and then decides. This type of decision‐making model was implied when researchers describe using feedback loops to communicate how they incorporated partner input and may be especially relevant for low‐stakes decisions.
- Consensus with input and acceptance from all team members. Overall, most examples discussed in the cases used this type of decision‐making model, especially for high‐stakes decisions such as study design.
- Consensus with a fallback, or a preset course of action if the team is unable to reach a consensus within an appropriate amount of time. Examples of consensus with a fallback include elevating decisions to leadership or agreeing to test out an approach; however, cases did not report agreeing to this preset course of action before the decision.
- Decentralized decision‐making, or delegating or assigning decisions to team members. Working groups with authority to make decisions for a specific task like recruitment is an example of this type of decision‐making model.

In addition to practices used by these cases to facilitate decision‐making (e.g., having a ground rule, behaviours to elicit and synthesize diverse viewpoints), study teams may consider additional practices to strengthen and improve the transparency of collaborative decision‐making processes. For example, as identified in other studies, at the start of working relationships, teams may want to work together to clarify roles, determine decision‐making approaches, define consensus and agree on a preset course of action if consensus cannot be reached.^18^, ^31^, ^32^

Perhaps most important, cases managed different viewpoints to meet the needs of their partners and the study, reflecting other literature that reinforces there is not a ‘one size fits all’ approach to engagement practices.^18^, ^33^ For example, in three cases, patient partners initially objected to randomization when an intervention perceived to be effective would not be available to all study participants. This finding reiterates other published literature, where possible study participants' willingness to undergo randomization drops as they learn about accumulating evidence of a treatment's effectiveness.^34^ In two cases, study teams identified alternate study design options that met researchers' criteria for rigour; in the third case, researchers and partners had a productive discussion about the importance of randomization, and partners agreed to continue with the randomized design.

### 4.1 Limitations and future research

The purposeful sampling approach of PCORI‐funded projects may limit the generalizability of the findings. The cases reflected a subset of projects from the original study on engagement's influence and impact, in which managing different viewpoints and priorities emerged as a challenge. Because we did not ask explicitly about these issues in the original study, other projects may have had similar experiences but did not disclose them. Also, although not a selection criterion, all cases in this study used collaborative decision‐making. Findings may not reflect projects using other decision‐making approaches or where collaborative approaches were not effective.

Further, for four of the six cases, more than 2 years had passed between these interviews and project completion. Interviewees may have not remembered all the strategies and behaviours used to manage different viewpoints and priorities. To enhance recall, interviewers prompted participants to remember specific events described in study progress reports or previous interviews. Finally, we did not conduct interviews with all partners involved in each project; findings may not represent all perspectives about what worked or did not work to balance viewpoints and priorities.

Even with these limitations, these findings provide a foundation for understanding how study teams manage and balance different viewpoints and priorities. Future research could explore how different decision‐making models affect researchers' and partners' perceived quality of engagement and decisions about study design and conduct. Studies could also examine relationships between decision‐making approaches and the ability to identify and work with partners from historically excluded and marginalized communities.

## 5 CONCLUSION

By illuminating how study teams can effectively manage different viewpoints and priorities, our findings can guide researchers and partners to plan and prepare for disagreements and collaborative decision‐making in ways that build trust and facilitate positive working relationships between and amongst researchers and stakeholders.

## AUTHOR CONTRIBUTIONS

All authors contributed to the design, data collection and analysis and writing or editing of this manuscript: Maureen E. Maurer co‐led conceptualization and design of the study, led the data analysis and wrote and revised initial and subsequent drafts. Tandrsssea Hilliard‐Boone contributed to the conceptualization and design of the study, conducted data collection and analysis and contributed to writing, reviewing and editing drafts. Karen Frazier contributed to the conceptualization and design of the study, conducted data collection and analysis and contributed to writing, reviewing and editing drafts. Laura Forsythe contributed to conceptualization and design of the study, contributed to data analysis and interpretation and reviewed and edited drafts. Rachel Mosbacher contributed to conceptualization and design of the study, contributed to data analysis and interpretation and reviewed and edited drafts. Kristin L. Carman contributed to the conceptualization and design of the study, contributed to data analysis and interpretation and reviewed and edited drafts

## CONFLICT OF INTEREST STATEMENT

The authors declare no conflict of interest.

## ETHICS STATEMENT

The American Institutes of Research Institutional Review Board reviewed this study.

## Data Availability

The data that support the findings of this study are available on request from the corresponding author. The data are not publicly available due to privacy or ethical restrictions.
